# Profiling of vaginal *Lactobacillus jensenii* isolated from preterm and full-term pregnancies reveals strain-specific factors relating to host interaction

**DOI:** 10.1099/mgen.0.001137

**Published:** 2023-11-27

**Authors:** Sai Ravi Chandra Nori, Tara K. McGuire, Elaine M. Lawton, Fionnuala M. McAuliffe, Douwe Van Sinderen, Calum J. Walsh, Paul D. Cotter, Conor Feehily

**Affiliations:** ^1^​ Teagasc Food Research Centre, Fermoy, Co. Cork, Ireland; ^2^​ APC Microbiome Ireland, University College Cork, Cork, Ireland; ^3^​ School of Microbiology, University College Cork, Cork, Ireland; ^4^​ SFI Centre for Research Training in Genomics Data Science, School of Mathematics, Statistics & Applied Mathematics, University of Galway, Galway, Ireland; ^5^​ UCD Perinatal Research Centre, School of Medicine, University College Dublin, National Maternity Hospital, Dublin, Ireland; ^6^​ Department of Microbiology & Immunology, Peter Doherty Institute for Infection & Immunity, University of Melbourne, Melbourne, Australia; ^7^​ Nuffield Department of Medicine, University of Oxford, Oxford, UK

**Keywords:** genomics, microbe-host interactions, *Lactobacillus jensenii*, preterm birth, vaginal microbiome

## Abstract

Each year, 15 million infants are born preterm (<37 weeks gestation), representing the leading cause of mortality for children under the age of five. Whilst there is no single cause, factors such as maternal genetics, environmental interactions, and the vaginal microbiome have been associated with an increased risk of preterm birth. Previous studies show that a vaginal microbiota dominated by *Lactobacillus* is, in contrast to communities containing a mixture of genera, associated with full-term birth. However, this binary principle does not fully consider more nuanced interactions between bacterial strains and the host. Here, through a combination of analyses involving genome-sequenced isolates and strain-resolved metagenomics, we identify that *L. jensenii* strains from preterm pregnancies are phylogenetically distinct from strains from full-term pregnancies. Detailed analysis reveals several genetic signatures that distinguish preterm birth strains, including genes predicted to be involved in cell wall synthesis, and lactate and acetate metabolism. Notably, we identify a distinct gene cluster involved in cell surface protein synthesis in our preterm strains, and profiling the prevalence of this gene cluster in publicly available genomes revealed it to be predominantly present in the preterm-associated clade. This study contributes to the ongoing search for molecular biomarkers linked to preterm birth and opens up new avenues for exploring strain-level variations and mechanisms that may contribute to preterm birth.

## Data Summary

Sequence data used in this study can be accessed from European Nucleotide Archive (ENA) under the accession number PRJEB34536. The genomes and metagenome assembled genomes generated in this study can be accessed from ENA under accession number PRJEB63084.

**Impact Statement**

The vaginal microbiome plays a crucial role in human health and has been shown to be predictive of adverse health outcomes such as preterm birth. Recent studies have associated *Lactobacillus jensenii,* a human vaginal commensal with preterm delivery. However most studies were limited to 16S rRNA sequencing. Here we used metagenomics and culturing techniques to generate high quality *Lactobacillus jensenii* genomes which enabled us to understand the strain-level genomic differences between full-term and preterm strains. We identified genomic signatures distinct to preterm strains and found potential gene clusters involved in glycogen utilization and microbe-host interactions. We showed the presence of preterm signatures in the global *L. jensenii* genomes which could be useful in developing biomarkers for early detection of preterm. Our work highlights the importance of strain-level insights in the vaginal microbiome field and contributed to on-going efforts of mechanistic understanding of the relationship between preterm birth and the vaginal microbiome.

## Introduction

The vaginal microbiome in humans is distinct, relatively less diverse than other body sites like the gastrointestinal tract [1] and often dominated by *Lactobacillus* species [2, 3]. By producing lactic acid, *Lactobacillus* spp. reduce vaginal pH and provide protection against the growth of pathogens [4–6]. Lactobacilli can also produce antimicrobials such as bacteriocins that benefit the host epithelium by maintaining membrane integrity [7, 8]. The species commonly found in the vaginal microbiota of healthy women are *Lactobacillus crispatus *, *Lactobacillus gasseri *, *Lactobacillus iners* and *Lactobacillus jensenii *; most of which produce large amounts of lactic acid [9–11] and are commonly categorised into community state types (CSTs) [2] CST-I, CST-II, CST-III and CST-V, respectively. The remaining CST, CST-IV, represents a community with reduced *Lactobacillus* and an increased collection of obligate and facultative anaerobes, including *Atopobium *, *Gardnerella *, *Prevotella* and *Megasphaera* [12], and has been linked to bacterial vaginosis (BV) [13]. Interestingly, CST-IV is also observed in a high percentage of the healthy population with asymptomatic BV. This suggests that the relationship between CST and BV is complex and likely involves other factors such as hygiene, ethnicity, and smoking [14]. Various epidemiological studies have associated mixed-species communities with an increased risk of poor health outcomes such as sexually transmitted infections [14, 15]. However, the mechanistic understanding of these correlations has yet to be explored. Furthermore, various studies have shown an increased risk of preterm birth (PTB) in women with BV [16, 17].

Approximately 15 million infants are born preterm (<37 weeks of gestation) annually, which is a risk factor for infant mortality and morbidity [18]. Several factors, such as maternal-fetal genetics, environmental interactions, and the vaginal microbiome, are important contributors to PTB [19]. Furthermore, bacterial infections such as urinary tract infections and periodontal disease have been associated with an increased risk of PTB [20, 21]. Previous studies have reported that the taxonomic composition of the vaginal microbiome has a notable influence on PTB risk [22–25]. Some previous studies have associated *Lactobacillus crispatus* with a lower risk of PTB [26]. However, a variety of different taxa have been associated with PTB in some studies [26, 27], whereas another study found no association between the vaginal microbiome and PTB [28].

A recent review by Gudnadottir *et al*. [29] suggested that analysing the vaginal microbiome could aid in predicting PTB. They linked *L. crispatus* dominance to a lower risk of PTB and *L. jensenii* abundance to a higher risk of PTB. Indeed, a study of 133 women, predominantly Caucasian, found a significant association between both *L. jensenii* and decreased lactate levels with PTB [30]. Recent reports also highlight that women with *L. jensenii* dominance over *L. crispatus* had lower lactate and glutamate levels in vaginal fluid and experienced more PTB [31, 32]. It is important to note that most studies were observational and based on 16S RNA sequencing, which limits the taxonomic resolution to the species level, at best, and further limits the potential to understand the functional potential of the community and investigate microbial-host interactions. Recent studies using shotgun metagenomics have begun to identify potential functional signatures associated with PTB, highlighting the power of this approach [26, 33]. However, in-depth mechanistic insights relating to strains and their functional differences are needed to identify potential molecular biomarkers that could aid in the early detection of PTB and perhaps reveal genes involved in important host-immune interactions.

In this study, we analyse data from shotgun metagenomics and genome-sequenced isolates to reveal strain-level variation among *Lactobacillus jensenii* from vaginal swabs of pregnant women associated with preterm and full-term birth, respectively.

## Methods

### Bacterial isolation and genome sequencing

Samples analysed in this study were previously collected as reported [26] with institutional ethics approval by the National Maternity Hospital Research Ethics Committee and maternal written consent. From the same swabs used for metagenomic sequencing, an aliquot of resuspension PBS was serial diluted and plated onto DeMan Rogosa Sharpe (MRS) agar and incubated anaerobically at 37 °C for up to 48 h. Colonies were subcultured onto MRS agar to ensure purity. The 16S rRNA gene from isolates was amplified by PCR using universal primers CO1 and CO2 [34] and Sanger sequenced to identify the species.

For whole genome sequencing, an overnight culture of an isolate identified by Sanger sequencing as *Lactobacillus jensenii* was used for extraction of genomic DNA using the MoBio PowerFood kit, following manufacturer’s instructions. DNA was subsequently quantified using Qubit HS DNA quantification kit and prepares for shotgun sequencing following Illumina Nextera XT protocol with sequencing on the Illumina NextSeq 500 platform.

### Genome assembly of cultured isolates

Adapter removal and quality filtering of the raw paired end fastq reads was performed using trim galore (v 0.6.6) [35], cutadapt (v 1.18) [36] and FastQC (v 0.11.9). The resulting filtered reads were assembled using spades (v 3.14.1) [37] with isolate mode and *L. jensenii* RefSeq genome (NZ_CP018809.1) was used as reference for assembly. The draft genomes were further scaffolded using online Medusa with RefSeq genome [38]. The quality of the genomes was determined using QUAST (v 5.1.0) [39].

### Reconstruction of metagenome assembled genomes

Metagenomic sequencing reads from the same samples cultured above and previously reported were used in this study [26]. Quality filtering and host contamination removal was done with read_qc module in the MetaWRAP (v 1.3.2) [40] using raw paired fastq reads. The assembly of contigs from the filtered reads were assembled using assembly module with metaspades option in the MetaWRAP pipeline. The assembled contigs were binned with the binning module with metabat2, maxbin2 and concoct options. The resulting bins were refined and reassembled with the filtered reads to improve the quality of the bins using bin_refinement and reassemble_bins modules in the MetaWRAP pipeline. The bins with completeness ≥90 % and contamination <5 % were considered high quality and retained for downstream analyses. The *L. jensenii* bins were identified with phylophlan (v 0.32) [41] using SGB.Dec20 database.

### Pangenome and phylogenetic analysis

The pangenome of *L. jensenii* genomes were reconstructed using roary (v 3.13) [42] with ‘-e –r –z’ parameters. The gene differences between fullterm and preterm genomes were identified using query_pan_genome script in the roary software. Genes associated with the preterm birth were identified using the Scoary script. Heap’s law was used to determine whether the pangenome is open (γ>0) or closed (γ<0). The core genome sequences from roary output were aligned with mafft (v 7.475) [43]. The resulting alignment was used for the reconstruction of maximum likelihood phylogeny using IQ-TREE (v 2.1.3) [44] with 3 000 bootstraps option. The output trees in the newick format were visualized using iTOL [45].

### Core genome variations and gene cluster analysis

Individual core gene families were extracted from the pangenome using roary multi_fasta script. The gene families were individually aligned with mafft (v 7.475) [43]. Using these sequences variations in the core genes between full term and preterm groups were identified with a custom python script. The genes were annotated with prokka [46] within the roary programme. The function of unannotated genes was further found using Eggnog mapper (v 2.1.7) [47] and online blast [48] against NCBI-NR database. The similarity and visualisation of the gene clusters were done using EasyFig (v 2.2.5) [49].

### Profiling of gene clusters in publicly available genomes

The publicly available 142 *L *. *jensenii* genomes were downloaded from the PATRIC database [50] on 25 April 2023. The high quality 63 genomes were filtered with >90 % completeness and <5 % contamination and only vaginal isolates. Average nucleotide identity was calculated with *L. jensenii* and *L. mulieris* RefSeq genomes using fastANI (v 1.32) [51] and ten *L *. *mulieris* genomes were removed resulting in 53 high quality *L. jensenii* genomes. The genes from the carbohydrate utitlisation and cell surface synthesis clusters were used as a database to identify the genes across the 53 *L *. *jensenii* using BLASTp (v 2.8.1) [48].

## Results

### Isolation and sequencing of *Lactobacillus jensenii*

Previously we have reported a functional difference in the vaginal microbiota composition of PTB women [26]. Here we used 89 vaginal metagenomic samples from 57 participants of the same cohort to reconstruct 11 high-quality (>90 % completeness and <5 % contamination) *L. jensenii* metagenome assembled genomes (MAGs). In addition, we isolated, and genome sequenced nine strains of *L. jensenii* from the same cohort, of which four were not paired to any MAG assembly (Table S1, available in the online version of this article). In total, 15 high-quality genomes (Table S2), nine from cultured isolates and six MAGs were used for analysis, with an isolate being considered over a MAG when both were recovered. Six of these genomes were from preterm pregnancies and the remaining nine were from full-term pregnancies. Of the six preterm samples, two were late preterm (36.4 and 36.7 weeks), and four were very preterm (28–32 weeks). The mean birthweight for preterm and full-term babies was 2 439 g and 4 061 g, respectively. Four women had a history of previous large loop excision of the transformation zone (LLETZ) surgery with two each in the preterm and full-term group.

### Pangenome and phylogenomic analysis reveals a distinct grouping of preterm and full-term *L. jensenii* genomes

**Fig. 1.**
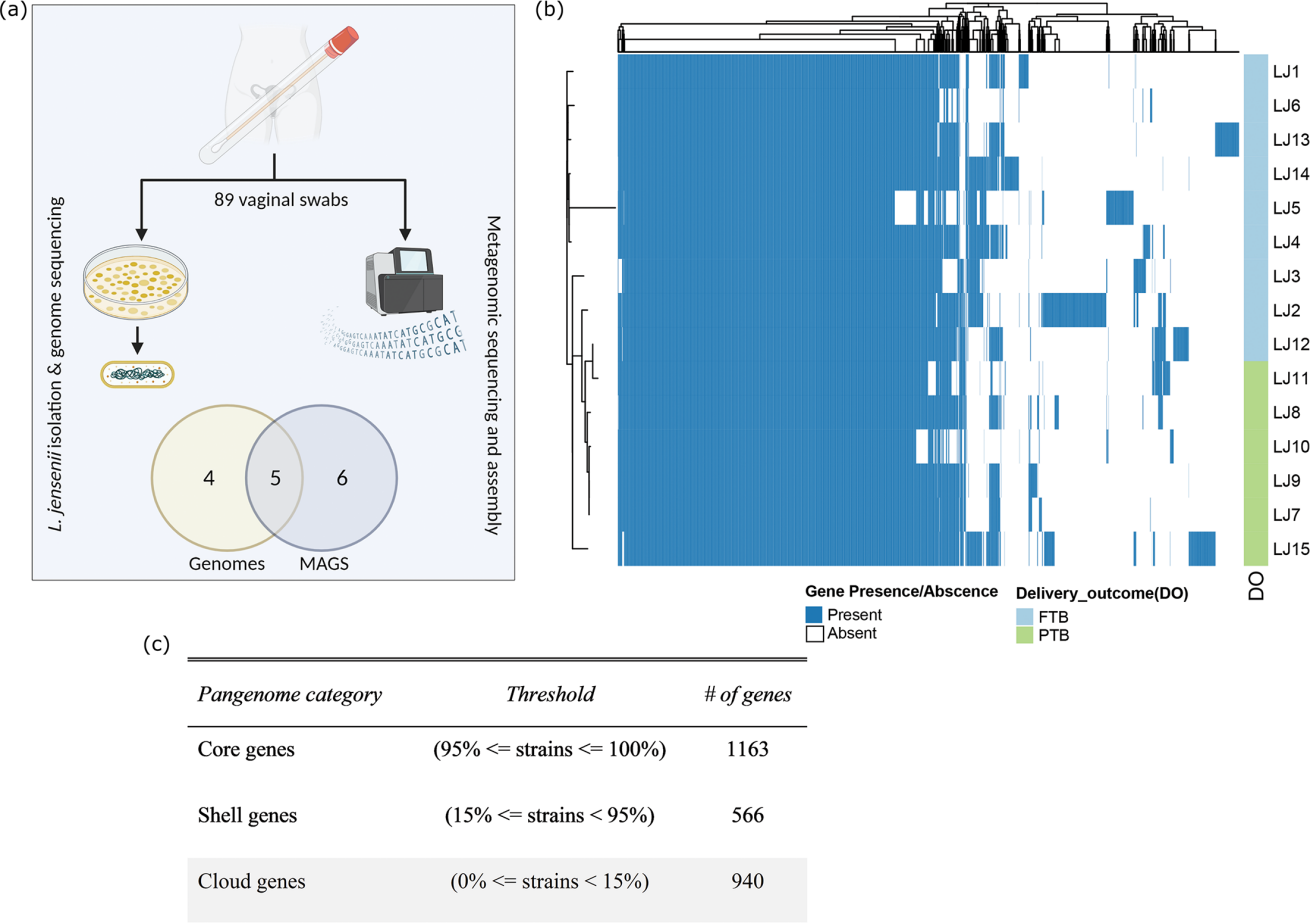
(**a**) Overview of *L. jensenii* sequenced from vaginal metagenomic samples in this study. The Venn diagram represents the number of genomes and MAGs recovered [Created with BioRender.com]. (**b**) Pangenome heatmap of *L. jensenii *. Rows are clustered based on the core genome identity, also represented by the phylogenetic tree on the left-hand side. For each gene, a blue cell indicates its presence, and a white cell indicates its absence in the corresponding genome based on the roary 95 % identity threshold. The legend shows the delivery outcome (DO) of each pregnancy associated with the genome. (**c**) Pangenome statistics of *L. jensenii* genomes in this study.

To facilitate an investigation of the genomic variation between preterm and full-term-associated *L. jensenii* strains, we performed a pangenomic reconstruction of the 15 *L *. *jensenii* genomes (Fig. 1b). A total of 24 928 genes were clustered into 2 669 unique gene families (Fig. 1c). Of the 2 669 gene families, 1163 were universally present (≥ 95 % strains) and classified as the core genome. The number of gene clusters in the shell and cloud genomes were 566 and 940, respectively. The pangenome analysis of *L. jensenii* revealed an open pangenome (Fig. S1).

**Fig. 2.**
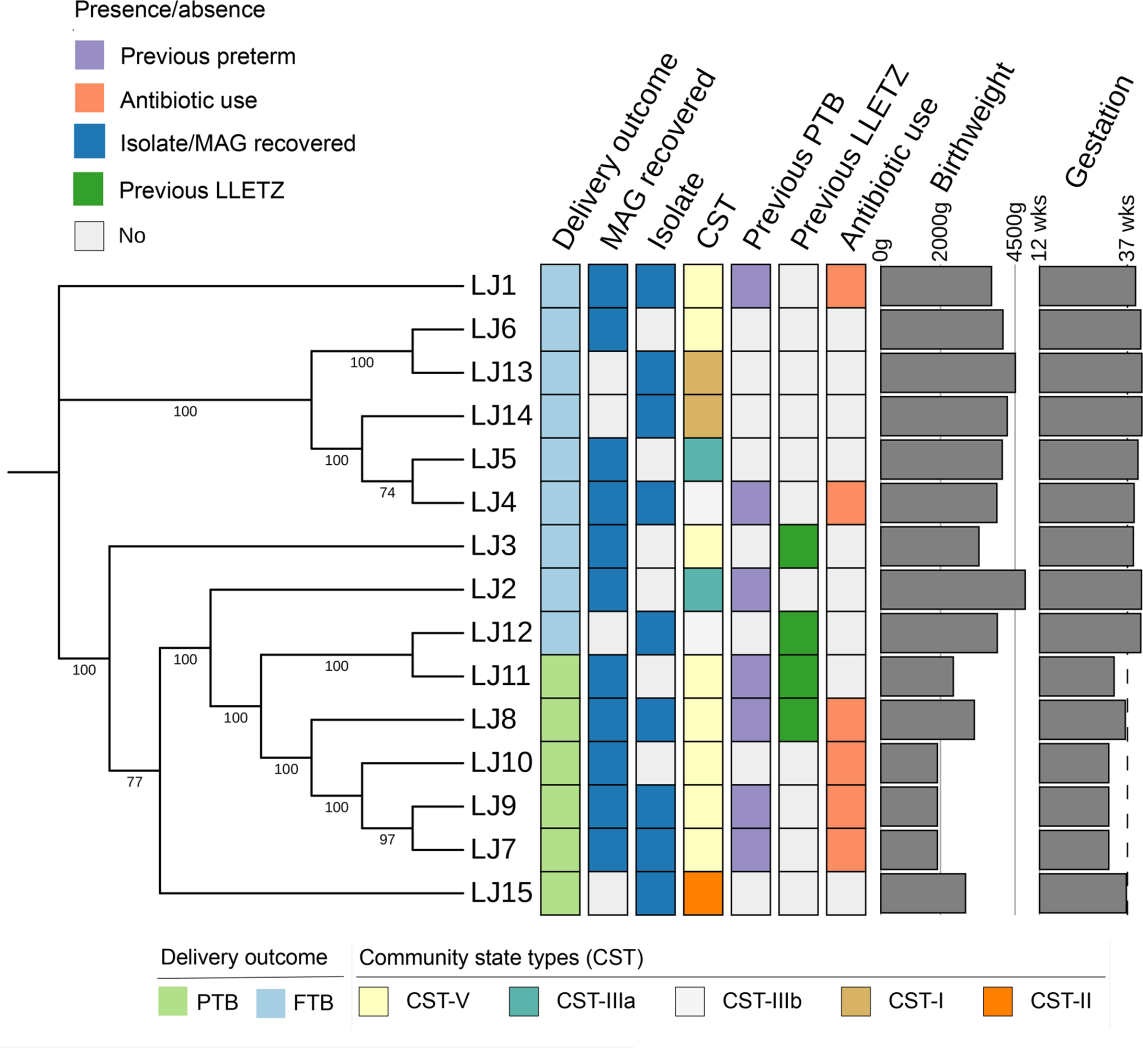
Maximum likelihood phylogenetic tree of the core genome of *L. jensenii *. Bootstrap values are represented on the branches. The heatmap on the right-hand side of the cladogram represents the delivery outcome, whether an isolate and/or MAG was recovered from this individual, community state type (CST), previous preterm birth status and LLETZ surgery, and antibiotic use in pregnancy. Bar plots show birthweight and gestation length.

Core genome phylogeny, as determined by SNPs in these core genes, revealed that strains from preterm group were distinct from full-term birth (FTB) (Fig. 2). However, three strains from the FTB group (LJ2, LJ3, LJ12) clustered within the PTB clade. Further investigation revealed that these strains were from women with previous LLETZ surgery and PTB history. Altogether, within the PTB clade, 6/9 strains were directly associated with PTB while the remaining three FTB-linked isolates were associated with a history of PTB or LLETZ surgery. Next, we performed an average nucleotide identity (ANI) comparison between the strains to further understand the genomic variation between these two clades. We found ANI values ranging from 99.30 to 99.91 suggesting a high level of genetic similarity with subtle strain-level variations (Fig. S2).

### Genetic variation and genes distinct to PTB associated *L. jensenii*

**Fig. 3.**
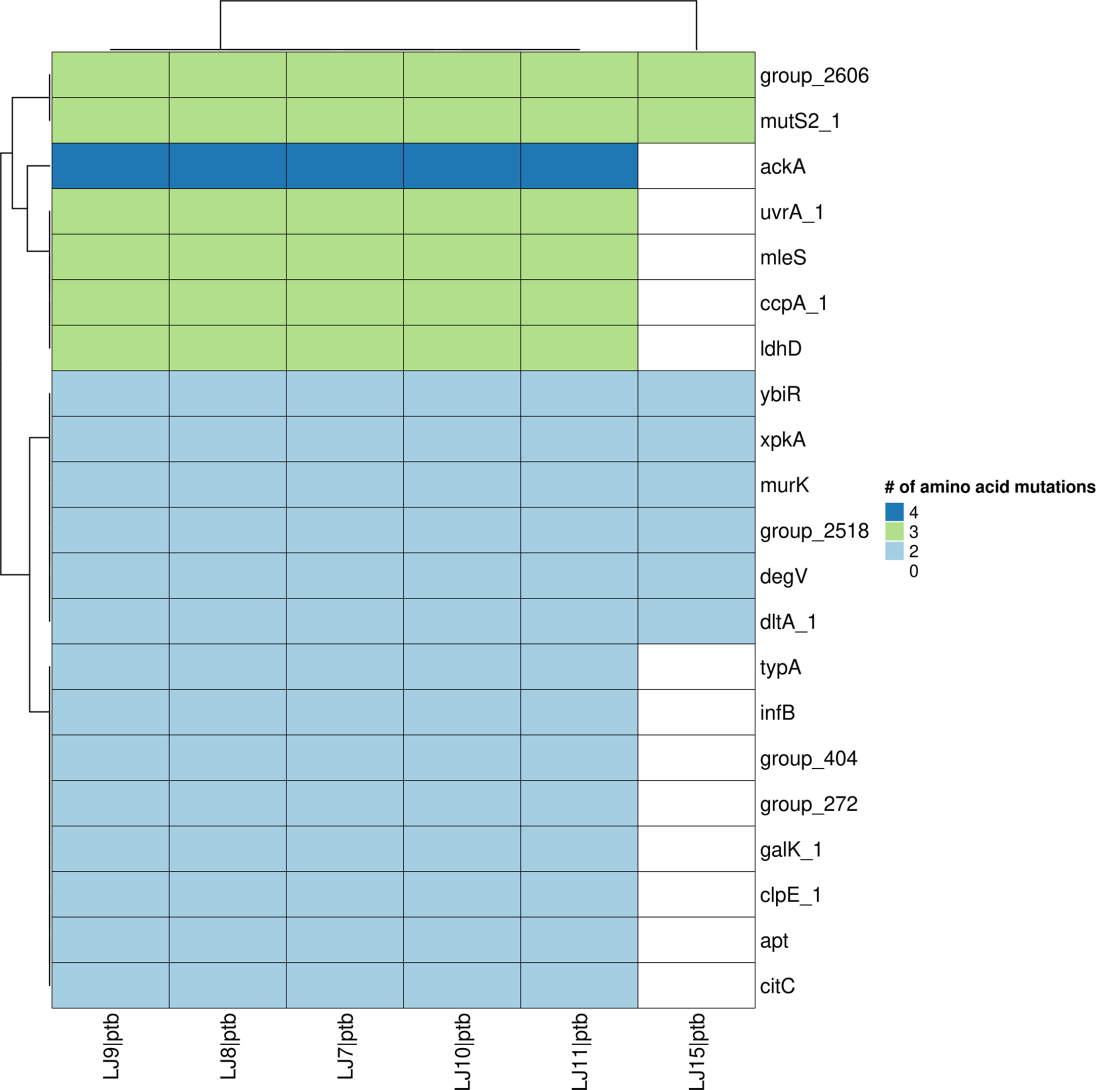
Heatmap showing distinct amino acid variations in preterm birth samples. Each colour represents the number of per-gene variations. Only genes with at least two variations were plotted (see Table S3).

A total of 1163 core gene families were extracted from the pangenome to identify variations in genes involved in core functions between the PTB and FTB strains. Following alignment of individual protein sequences from these gene families, 185 non-synonymous mutations specific to PTB genomes were identified in 156 core genes (Table S3). A notable number of differences between PTB and FTB strains were observed in genes involved in lactate metabolism (*ldhD*), acetate metabolism (*ackA*), and cell wall-related functions (*murK* and *ycbB*) (Fig. 3), revealing a genetic signature distinct to PTB associated *L. jensenii *.

Using roary, we analysed the genomes of *L. jensenii* to determine if further genetic differences between strains were present. Genes distinct to preterm/full term birth were identified using the whole pangenome including cloud and shell genes. However, we found significant genes only within the core genome. In total we observed 14 genes that were distinct to PTB strains and four genes distinct to FTB strains. Fifty percent (7/14) of these had an unknown function following automated annotation with Prokka. Supplemental annotation with Eggnogmapper and BLASTx against the NCBI-NR database identified the functions of 85 % (12/14) of these genes. The PTB specific genes were present within two gene clusters harbouring putative signal peptide, glycosyl transferases, polysaccharide synthesis genes and glycosyl hydrolases (Table S4).

### Preterm birth associated *L. jensenii* possess a gene cluster with a predicted role in cell surface proteins synthesis

**Fig. 4.**
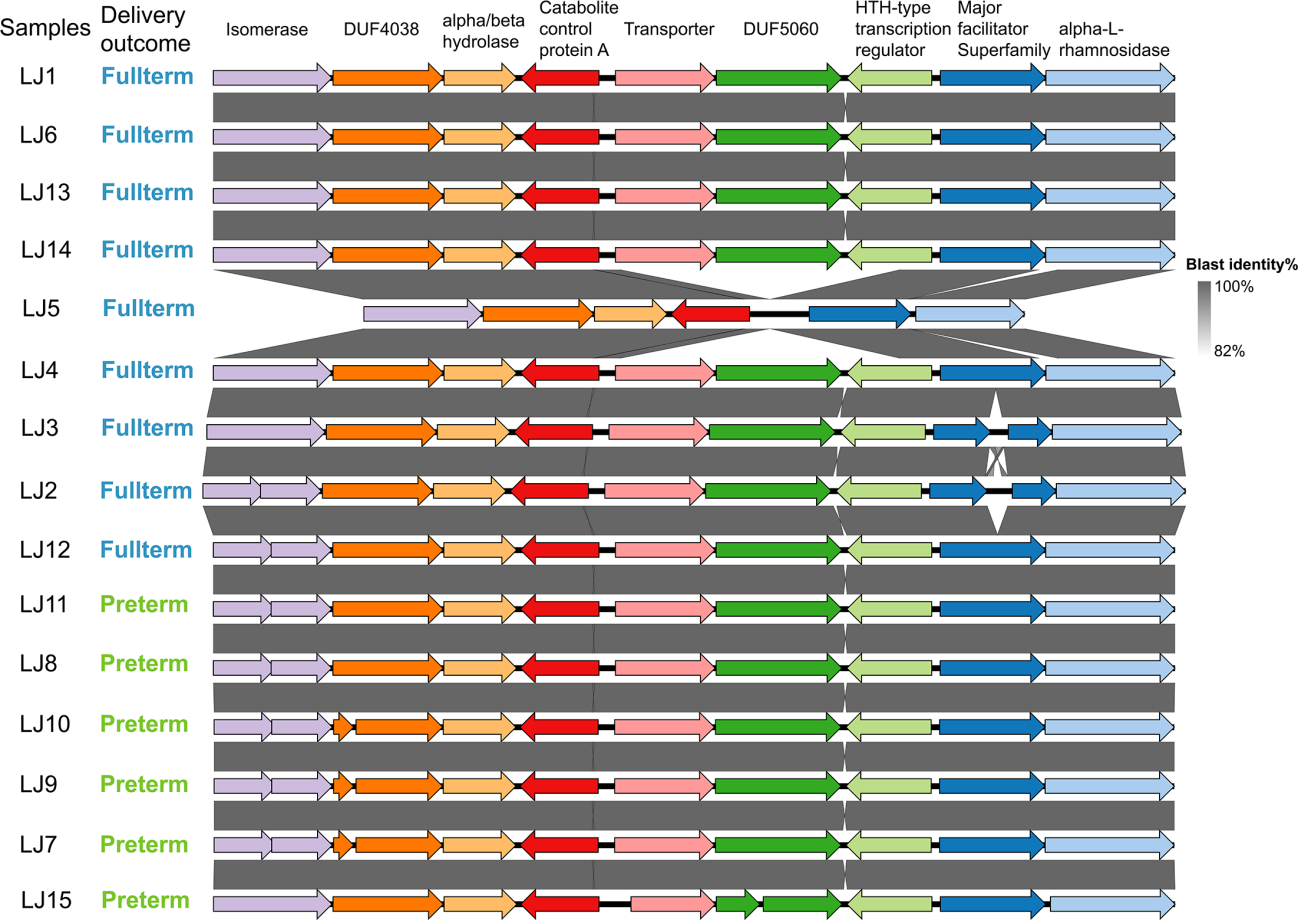
Schematic overview of the organisation and sequence relatedness of the glycan utilisation gene cluster of *L. jensenii *. The homology of each gene is represented by different shades of white (low homology) to grey (high homology) between each genome. The colour of the arrows represents each gene.

A gene cluster of ten genes distinct to preterm strains that were identified in the pangenome analysis was observed in a region adjacent to a further five genes that were also present in full-term genomes (Fig. 4). The gene cluster harbours a large genomic region of ~10 kb identified as a LPXTG-motif cell wall anchor (CW_MucBP). Within this is a putative single coding sequence for a domain of unknown function (DUF285), a mucin binding domain (MucBP) and a YSIRK signal motif. In addition, this gene cluster contains several putative glycosyl transferase genes (GT), including GT1, GT2 and GT4. Further, a Flippase (MurJ) was also found in the gene cluster. This gene cluster is predominantly found in genomes from preterm samples. A strong association with the preterm trait was observed for eight out of the ten genes. (Table S4). In full-term isolates, the genes required to synthesise polysaccharides, cell surface proteins and mucosal adhesion were absent (Fig. 4).

### *L. jensenii* possesses a gene cluster with putative glycan utilisation genes

**Fig. 5.**
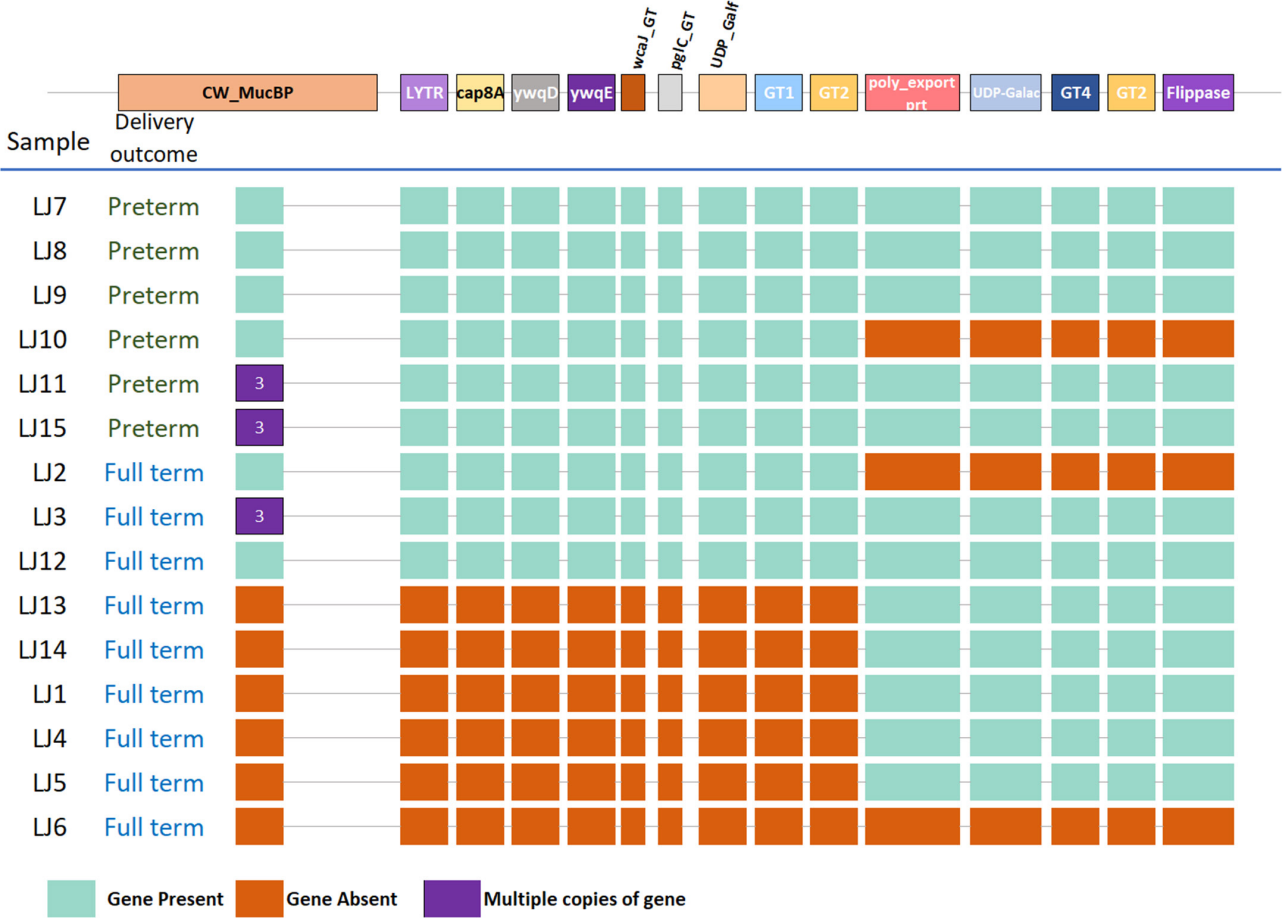
Schematic overview of the organisation of a putative cell surface glycan gene cluster of *L. jensenii *. The length of this gene cluster is ~20 kbp. The colour of each box indicates the presence (mint green) and absence (orange) of the corresponding gene annotated across the top.

We identified a gene cluster containing putative glycosyl hydrolase genes from the roary analysis within all genomes of *L. jensenii *. This cluster was approximately 11 kbp in length and comprised of genes predicted to encode an alpha-rhamnosidase, membrane transporters, an isomerase and two other genes of unknown function, designated DUF4038 and DUF5060 (Fig. 5). Literature searching suggested that DUF4038 and DUF5060 genes are putative glycoside hydrolase and glycoside hydrolase-associated C-terminal with the closest homologs to GH13 and GH14 family, that is, alpha-amylase and beta-amylase [52]. In addition, the alpha-beta hydrolase fold with potential serine protease function and major facilitator superfamily (MFS) transporters along with two potential GHs belonging to GH1 and GH28 were also identified flanking this region. While this cluster was conserved between all genomes, the putative isomerase gene contained a frameshift mutation that was absent in 5/6 PTB genomes and 2/9 FTB genomes, resulting in a predicted change in coding sequence for these strains. Similarly, the DUF4038 gene also has large deletion in 3/6 PTB strains, but none of the FTB genomes.

### Profiling of preterm associated gene clusters in publicly available *L. jensenii* genomes

**Fig. 6.**
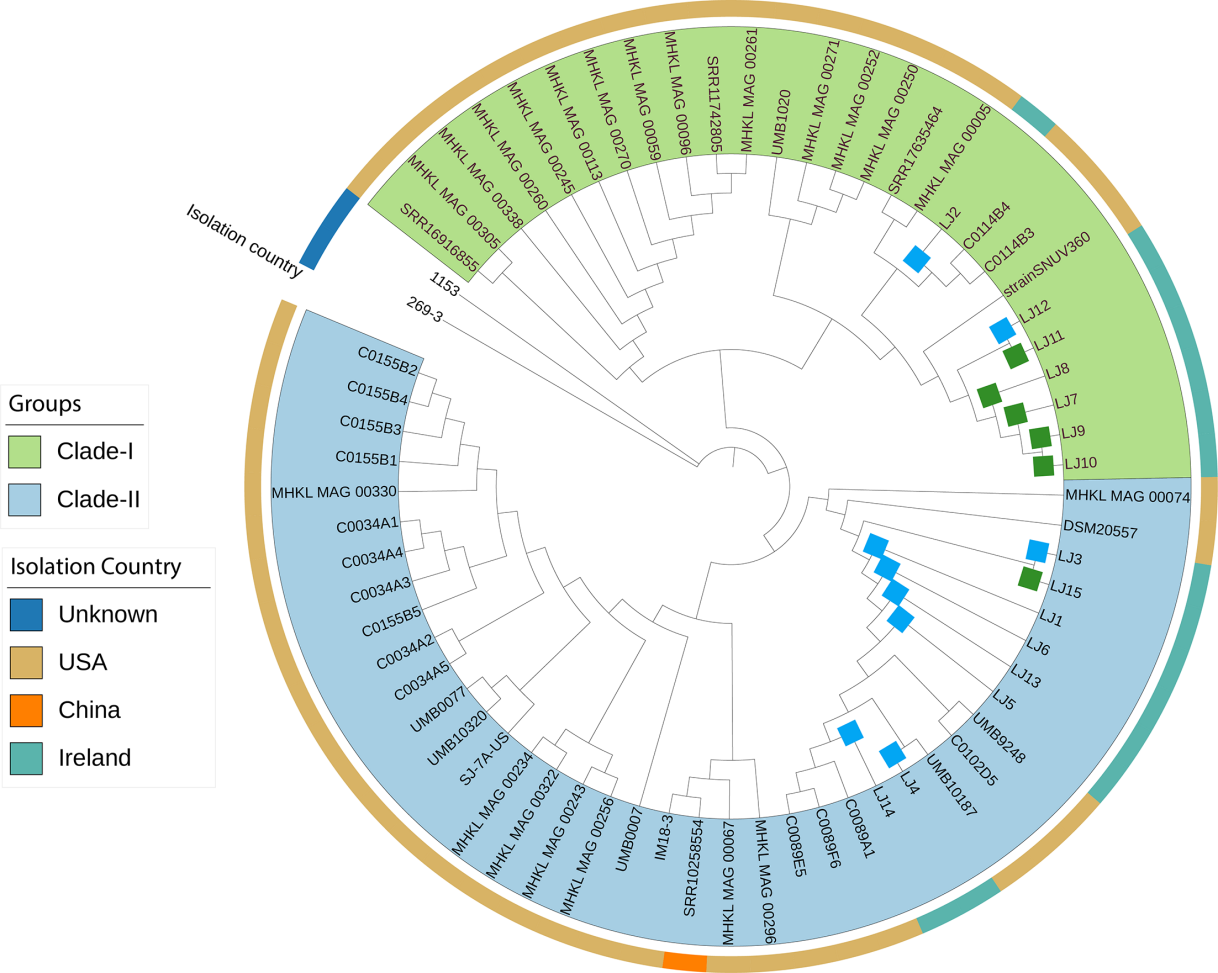
Maximum likelihood phylogenetic tree of *L. jensenii* species reconstructed with publicly available genomes. The outer circle represents the isolation country. The green colour on labels indicates Clade-I and blue indicates Clade-II. The green squares on the branches represent preterm isolates and blue squares indicates full-term strains from our dataset.

**Fig. 7.**
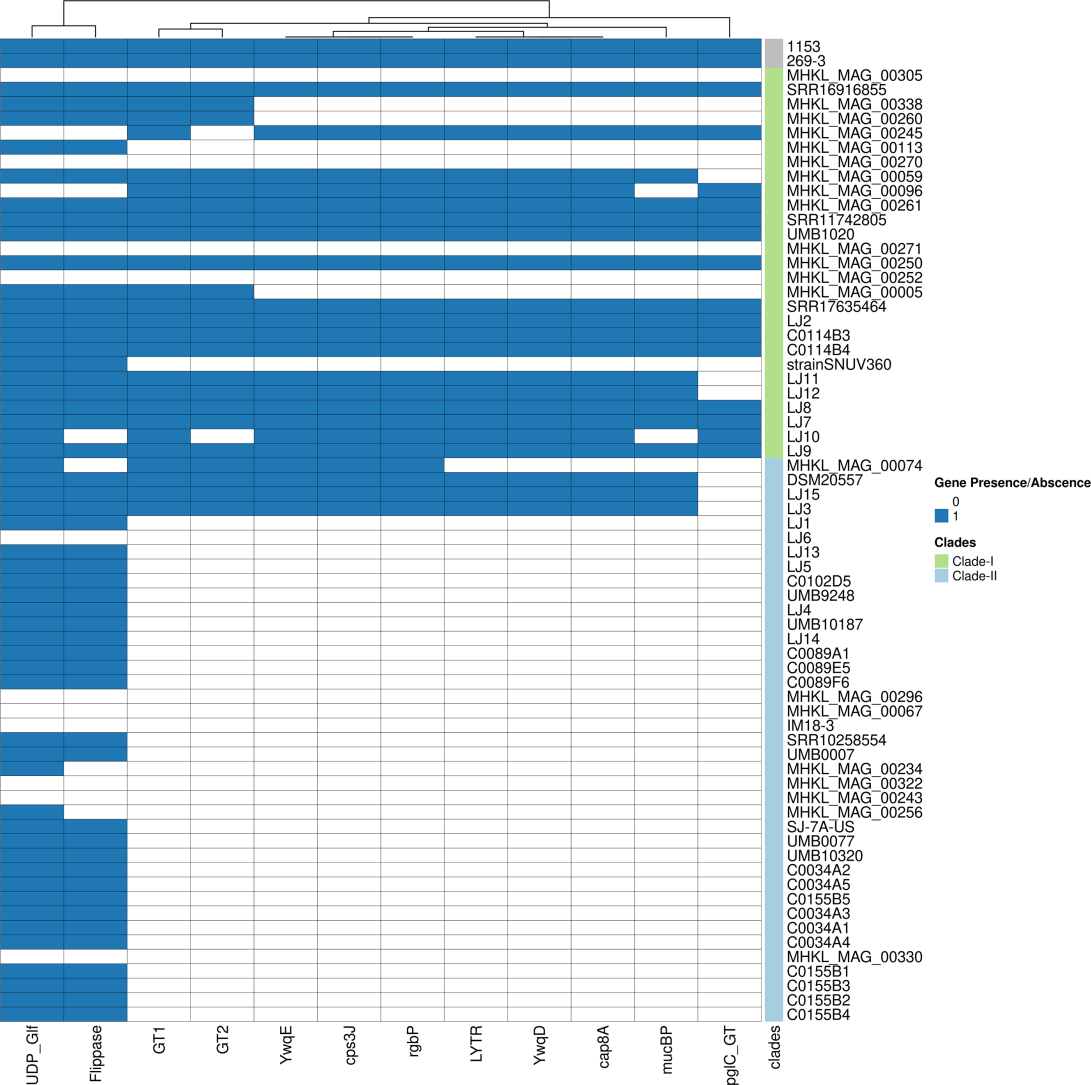
Heatmap showing the presence and absence of genes from the putative cell surface protein encoding cluster identified in preterm strains. The x-axis represents the genes from the gene cluster. For each gene, a blue cell indicates its presence, and a white cell indicates its absence in the corresponding genome. The bar on the right indicates the clades respective to the core genome phylogeny.

To identify the phylogenetic patterns and presence/absence of preterm associated genomic signature across *L. jensenii* species, we used publicly available genomes along with the genomes from our study. A very high ANI comparison value was observed for all genomes ranging from 98.98 to 100 (Fig. S4). The core genome phylogeny of these genomes revealed two distinct clades in the *L. jensenii* species (Fig. 6). The preterm and full-term related genomes from this study mainly clustered in two different clades, i.e. Clade-I and Clade-II, respectively. Genes from the putative carbohydrate utilisation gene cluster were present in all the isolates from both clades (Fig. S3). The putative cell surface protein encoding gene cluster was found to be predominantly present in the same clade as the preterm-associated genomes (Clade-I) (Fig. 7).

## Discussion

Fifteen high-quality *L. jensenii* genomes were analysed from vaginal samples from six preterm and nine full-term pregnancies using a combination of metagenomic sequencing and targeted isolation. In-depth genomic analysis revealed a distinct phylogenetic clade of preterm genomes and identified differences in the core genome for genes involved in cell wall formation and the metabolism of lactate and acetate. Further pangenome analysis identified a gene cluster predicted to be involved in the degradation of glycans typical to vaginal tract including glycogen. Another gene cluster specific to PTB strains was identified with a potential role in the synthesis of cell surface proteins which are crucial for microbial-host interactions. In addition, we profiled the presence of these gene clusters in publicly available genomes, which revealed cell surface protein synthesis genes to be predominantly present in the preterm-associated clade. These results suggest that the preterm *L. jensenii* group carrying these gene clusters may have a functional advantage through an ability to utilise various sugars and potential host-immune interactions within the vaginal niche. The data presented here also makes a significant contribution to vaginal *L. jensenii* reference genomes. Through detailed strain-level analysis, we compared full-term and preterm strains highlighting key genomic variations with a potential role in preterm complication.

Changes in the composition of the vaginal microbiota can be influenced by several factors, such as changes in host physiology, unprotected intercourse, or hormonal fluctuation [53, 54]. This makes it challenging to develop biomarkers for preterm/dysbiotic conditions. Recent studies have shown vaginal metabolites and microbiome profiles could be useful in predicting preterm [55, 56]. However, the mechanistic understanding of vaginal microbial genomic factors is needed to develop standard molecular based biomarkers that could aid in early PTB detection. A recent study has demonstrated that crosstalk between microbial recognition mediators and host immunity could play a key role in preterm complications highlighting the role of microbial cell surface components in host-immune modulation [57]. However, the role of microbial cell surface proteins, particularly glycans that can interact with host epithelial cells, has been poorly studied in vaginal microbial settings compared to the gut microbiome [58]. This is due to a majority of studies being limited by a reliance on 16S rRNA sequencing. As such, detailed strain-level genomic insights are lacking in the field. Despite this, recent studies have shown that some *Lactobacillus crispatus* strains with pullulanase type I gene (*pulA*) can directly degrade glycogen [59, 60]. It is also reported that the *L. crispatus* strains isolated from dysbiotic vaginal microbiota have a distinct glycosyltransferase gene that could play a role in cell surface glycome [59], further highlighting the potential importance of strain-level differences in vaginal bacteria.

Here we report two distinct gene clusters in *L. jensenii* with one predicted to have a diverse carbohydrate utilisation potential and another cluster involved in the biosynthesis of cell surface glycans with a potential role in preterm complication. The ability to selectively use various carbon sources such as glycogen and mucins can be beneficial for *L. jensenii* to survive in the fluctuating vaginal environment. Glycogen produced by vaginal epithelial cells is considered a nutrition source for vaginal bacteria [61], but is thought to be dependent on the initial activity of host amylase to facilitate subsequent use by vaginal commensals [62]. We identify the presence of probable alpha/beta-amylase genes, GH13/GH14 (DUF4038), in a glycan utilisation gene cluster along with a gene predicted to encode an alpha-l-rhamnosidase, which may be involved in the hydrolysis of α-linked l-rhamnosides from polysaccharides. This suggests *L. jensenii* might have the ability to utilize distinct carbon sources. Notably, a recent metatranscriptomics study [63] identified GH13 and glycosyl-transferase (GT) 35 as active glycogen-debranching genes across various CSTs. Additionally, the identified variations in DUF4038 (GH13) and *ccpA* genes in the preterm strains relative to the full-term strains indicate a functional difference in metabolic pathways for carbohydrates. Previous studies have shown subtle genetic variations in the glycan utilisation genes can have a strong impact on the phenotype of the strain [59].

In the glycan utilisation gene cluster, we have identified a fucose isomerase gene along with alpha-beta hydrolase fold with a potential serine protease flanking this region. It is possible that *L. jensenii* can use this gene machinery for adhesion to cervical mucus present in the vaginal epithelium [64, 65]. As the viscoelastic structures of mucins are the stiff arrangement of carbohydrate side chains with serine-threonine peptide backbone, production of a serine protease may help reduce the viscoelasticity [65] and enhance adhesion. Interestingly, we have identified an MFS transporter, two amino acid permeases and two GHs belonging to potential GH1 (β-glucosidase/ β-galactosidase) and GH28 (polygalacturonase) groups flanking this gene cluster. As galactose is a common component of mucins, this suggests that *L. jensenii* may utilise sugars from mucin oligosaccharides for nutrition [63]. Critchfield *et al*. [66] showed that cervical mucus from women at high preterm risk has more extensible and weaker gels than low-risk women, suggesting that alterations to surface mucus may contribute to PTB.

Microbial cell surface proteins are crucial to interactions with vaginal epithelial cells for various functions from commensalism to invasion [67]. However, the microbial cell surface glycome in the vaginal microbiome is poorly studied particularly in the vaginal commensals like *L. jensenii *. A recent study has identified a distinct glycosyltransferase in a gene cluster in *L. crispatus* from a dysbiotic vaginal microbiota suggesting the possibility of a distinct microbial cell surface glycome in vaginal bacteria from dysbiotic environments [59]. In this study, we report a distinct gene cluster in vaginal *L. jensenii* containing genes involved in the biosynthesis and export of cell surface proteins with a potential role in microbial-host interactions. We found *cap8A*, *ywqD* and *ywqE* homologues in this cluster, which have been reported to have a direct role in the polysaccharide production in *L. lactis* and *Bacillus subtilis* [68, 69]. The presence of potential diverse glycosyl transferases indicate the role of this gene cluster in glycan synthesis. We found a YSIRK signal motif which is associated with the efficient translocation of the surface protein into cellular membranes for anchoring or secretion from the cell [70]. Moreover, the presence of a flippase, known to have a key role in export of lipid bound oligosaccharides in *E. coli* [71], provides further evidence that this gene cluster is involved in the synthesis and translocation of glycans to the cell surface. The fact that these genes are associated predominantly with preterm strains indicates the potential for a difference in the cell surface glycome between preterm and full-term strains, which could have implications for immune evasion.

Further, core genome phylogeny revealed clustering of preterm and full-term strains into two different clades indicating genomic variations distinct to the preterm group. Although the PTB clade did contain three genomes from full-term deliveries, we noted that one of these genomes was from a woman with a previous PTB, and others had previous LLETZ surgery, both risk factors in themselves for PTB. Moreover, we identified variations in the core genes, notably in the acetate, lactate and cell wall related functions in preterm group. Similarly, we have also identified these gene variations in the publicly available *L. jensenii* genomes (Table S5). Taken together this pattern raises the possibility that host vaginal conditions could select for different strains of *L. jensenii *. The largest number of non-synonymous mutations were present in an essential acetate metabolism gene, acetate kinase (*ackA*). In *Escherichia coli,* it has been shown that deletion of *ackA* led to severe growth impairment by the reduction in glucose uptake rate and concentrations of ATP generated, highlighting the importance of this gene [72]. Notably, a study of 82 pregnant women showed that levels of cervicovaginal fluid acetate were predictive of preterm outcome [73]. Another study with a Caucasian population reported that *L. jensenii* dominance and decreased lactate levels were observed in women who delivered preterm [30]. In preterm strains, we identified three variations in lactate dehydrogenase (*ldhD*), a key gene responsible for lactic acid production. In addition, other mutations are found in core genes involved in the regulation of glycosyl hydrolases (*ccpA*), such as β-glucosidase and β-galactosidase and bacterial genetic diversity (*mutS2*) [74, 75]. Taken together, these results suggest a functional difference between full-term and preterm strains including lactate and acetate metabolism that may play a role in vaginal homeostasis.

The findings of this study provide insights relating to strain-level genomic signatures of *L. jensenii* from preterm and full-term isolates, which could be useful in developing biomarkers of PTB. Possessing the genetic machinery to ferment various carbohydrates along with adhesion genes can be beneficial for *L. jensenii* to survive in conditions associated with preterm birth and the presence of different cell surface synthesis genes may even be indicative of distinct microbial-host immune interactions in preterm strains. The high-quality genomes generated in this study enabled us to identify the subtle genomic differences in preterm and full-term groups. However, limitations exist due to the relatively small number of vaginal *L. jensenii* genomes and MAGs associated with PTB. Further studies with larger samples coupled with *in vitro* studies are needed to validate the implications of these genomic variations to better understand *L. jensenii'*s role in preterm.

## Supplementary Data

Supplementary material 1

Supplementary material 2
