# Supplementary material for: Profiling of vaginal Lactobacillus jensenii isolated from preterm and full-term pregnancies reveals strain-specific factors relating to host interaction
Source: Microb Genom. 2023 Nov 27;9(11):001137. doi: 10.1099/mgen.0.001137 (PMC10711310; doi:10.1099/mgen.0.001137)
Supplement: Supplementary material 1 [file mgen-9-1137-s001.pdf]

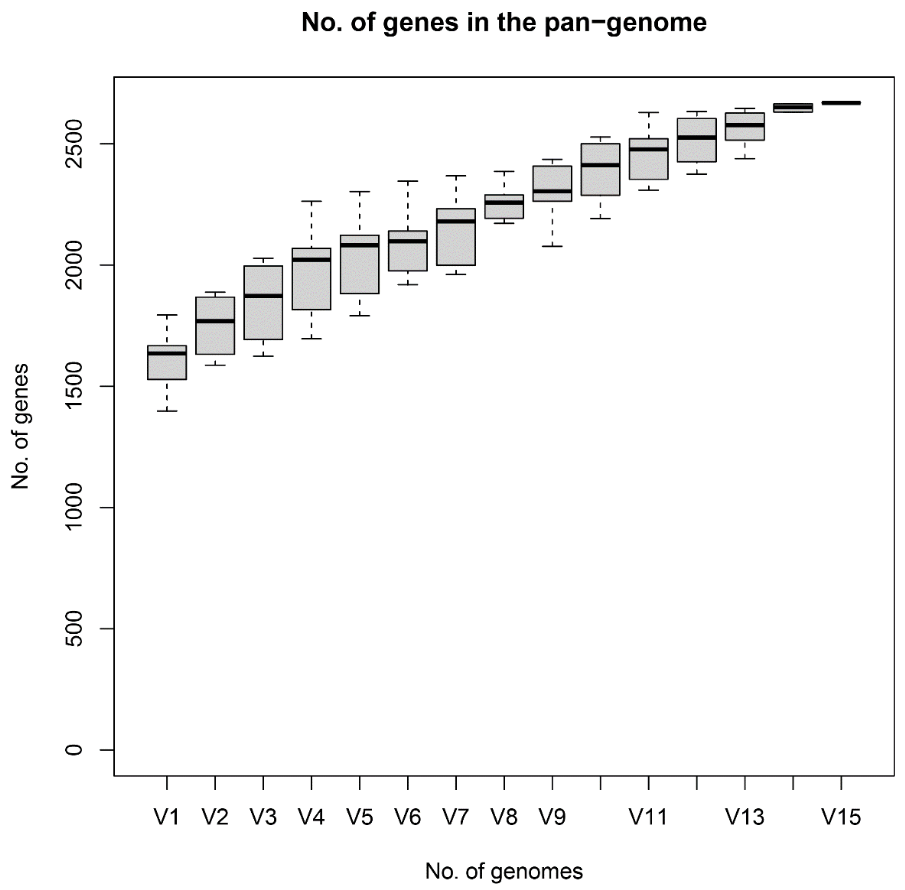

Supplementary. Fig. 1. Plot showing the number of genes (y-axis) in each of the *Lactobacillus jensenii* genomes (x-axis) generated in our study indicating an open pangenome with size increasing with every new genome added.

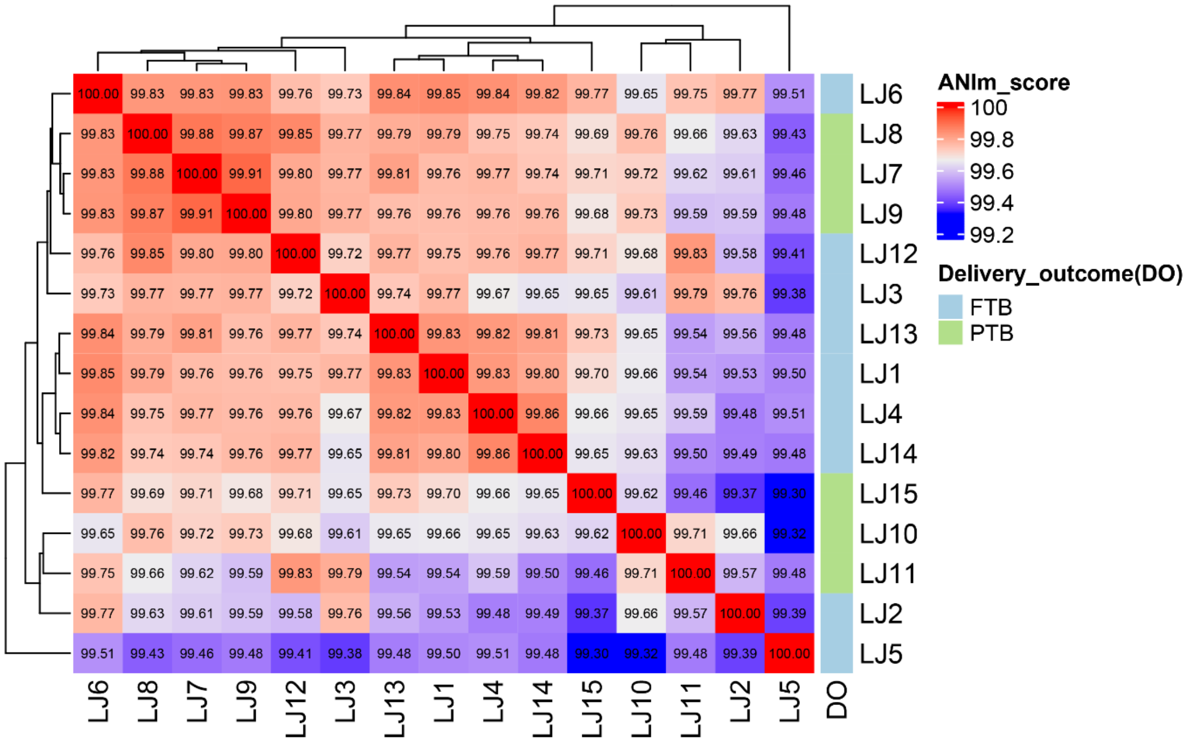

Supplementary. Fig. 2. Average Nucleotide Identity (ANI) heat-map showing the ANI scores between 15 *L. jensenii* in our dataset and side bar representing the delivery outcome (DO) of each pregnancy associated with the genome.

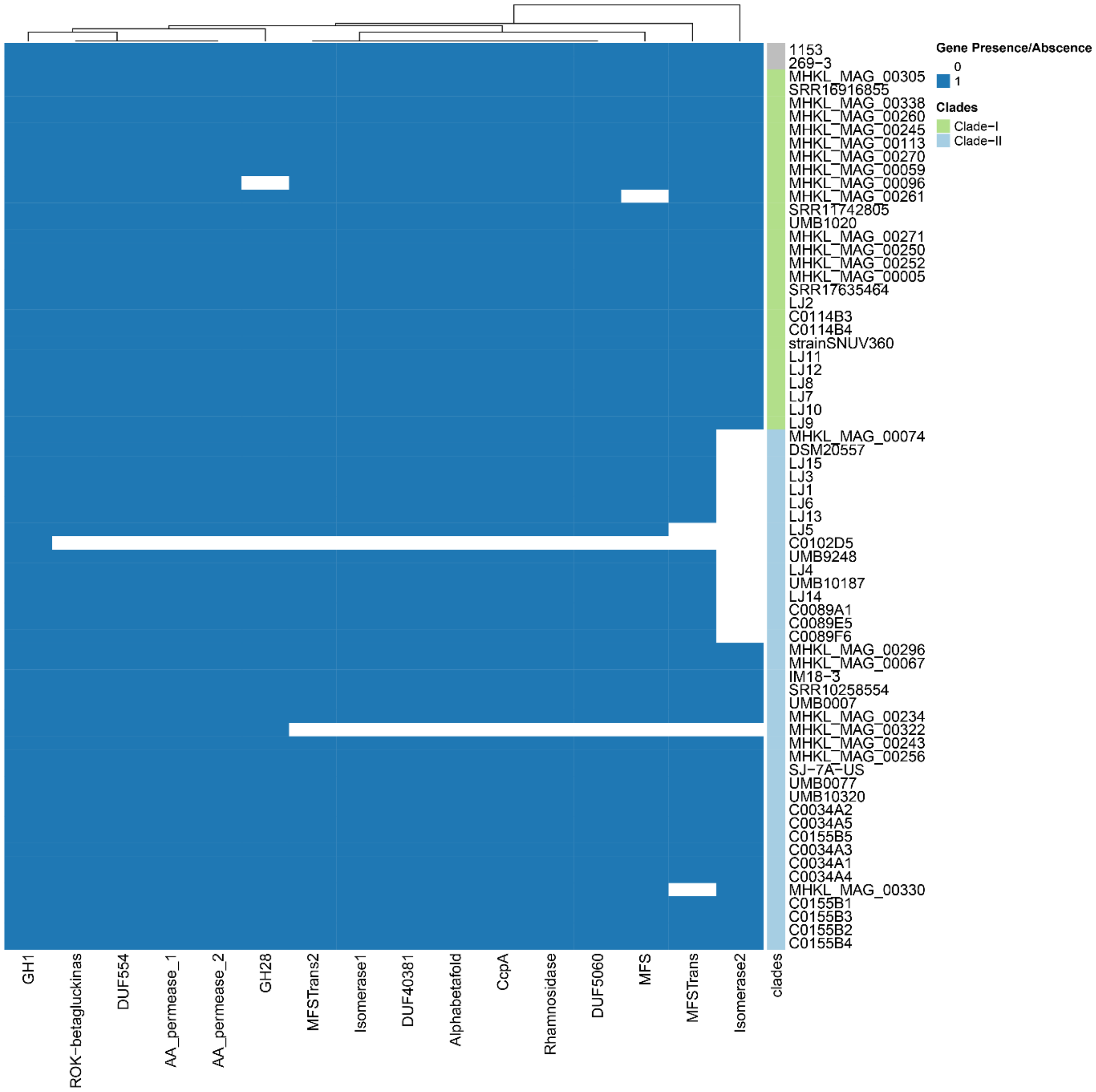

Supplementary. Fig. 3. Heatmap showing the presence and absence of glycan utilisation cluster genes in the publically available genomes. The bar on the right represents the clades respective to core genome phylogeny. The green represents the preterm associated clade, blue represents the full-term clade and grey indicates unknown.

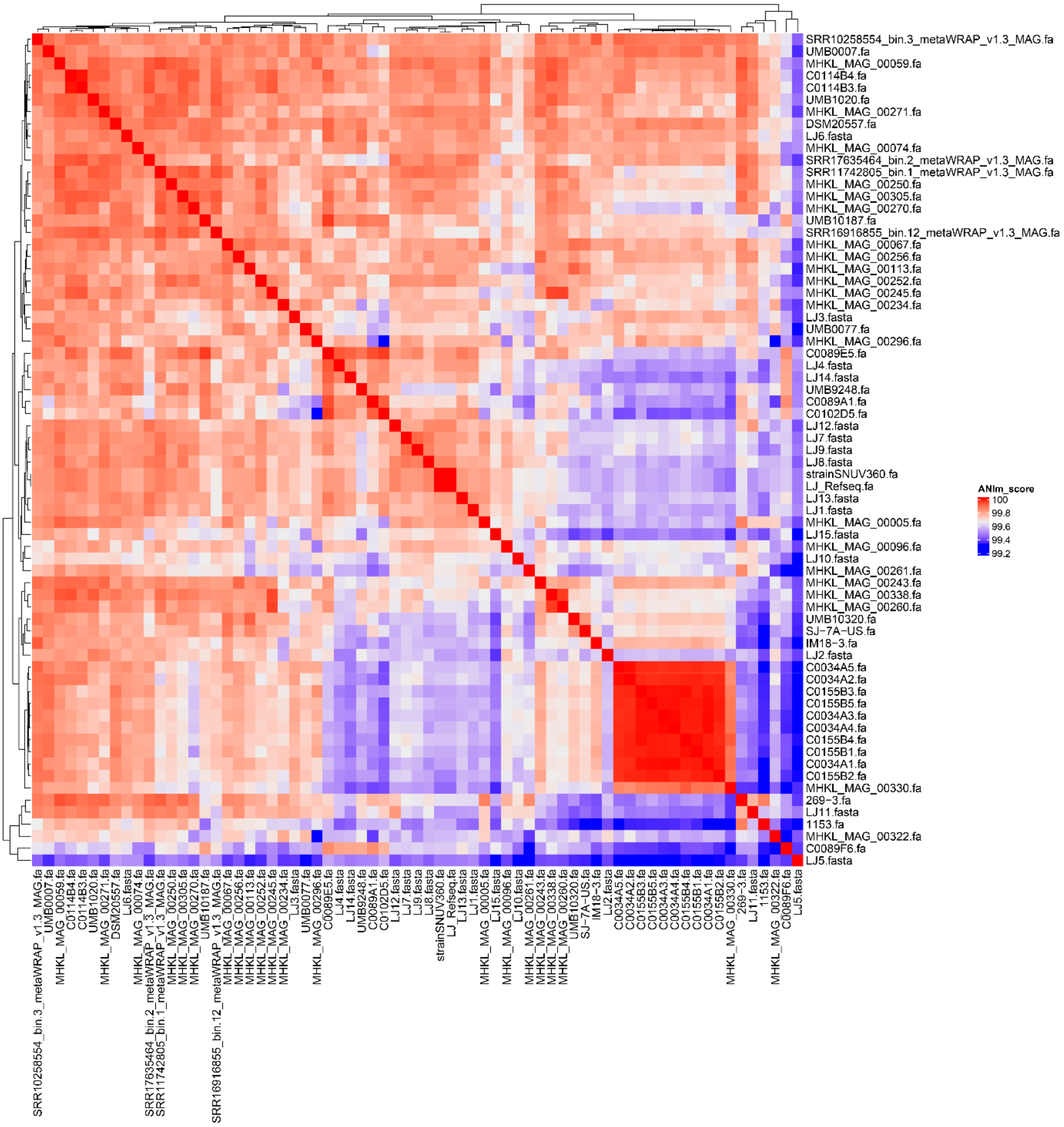

Supplementary. Fig. 4. Average Nucleotide Identity (ANI) heat-map showing the ANI scores between 15 *L. jensenii* in our dataset along with publically available genomes.
